# 1887. Deep Spatial Characterization of Tertiary Lymphoid Structures in Pulmonary Tuberculosis Granulomas

**DOI:** 10.1093/ofid/ofad500.1715

**Published:** 2023-11-27

**Authors:** Emmanuel Ogbonna

**Affiliations:** Harvard Medical School, Boston, Massachusetts

## Abstract

**Background:**

Tuberculosis is a multi-pronged public health menace responsible for greater global mortality than any other bacterial disease, causing around 1.6 million deaths yearly. Efforts to eliminate TB are complicated by antibiotic resistance, variable vaccine efficacy and insufficient understanding of its complex underlying biology. Lung granulomas are lesions central to TB pathobiology, immune response, disease progression, and treatment. They aid in *Mycobacterium tuberculosis* containment and clearance but paradoxically contribute to pathogen persistence. Concerted research efforts are currently channeled towards granuloma resolution by the host immune system. Tertiary lymphoid structures (TLSs) are ectopic lymphoid formations which can organize around granulomas and supply B- and T-cells, dendritic cells, etc., to infiltrate the core. Prior studies have shown a correlation between the presence of TLSs and increased protection against *Mtb* infection.

**Figure 1.**
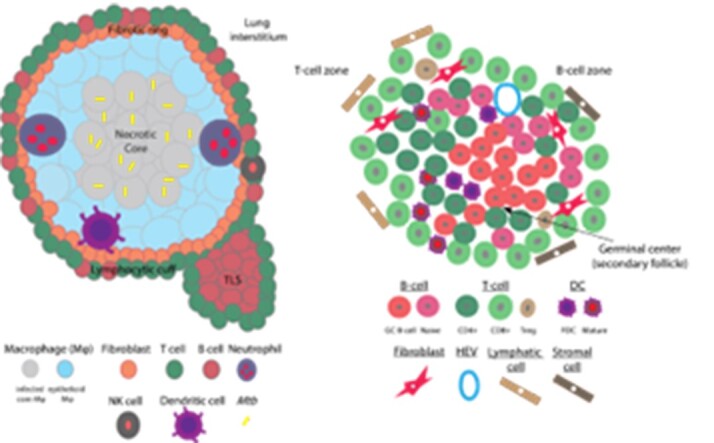
TB granuloma microenvironment architecture. A. Cartoon showing TB granuloma within infected lung tissue. B. Model of the composition of a TLS. Cells are segregated into T- and B-cell zones. High endothelial venules aid extravasation of immune cells from the blood (DCs, T and B cells, and Tregs). B cells can cluster in follicles with actively replicating B-cell germinal centers around follicular dendritic cells

**Figure 2.**
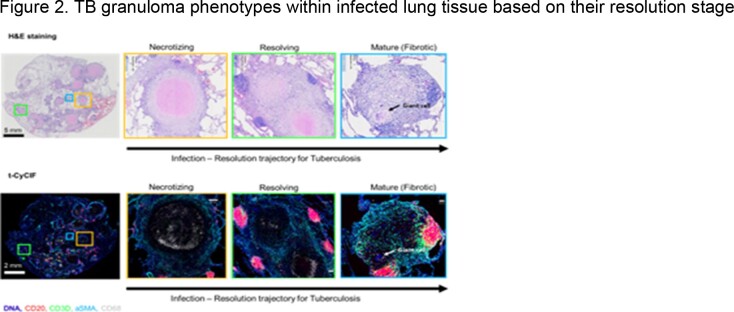
TB granuloma phenotypes within infected lung tissue based on their resolution stage A. H&E staining showing tissue pathology B. t-CyCIF. This preliminary multiplexed image data from a single patient resection shows heterogenous granulomas with TLSs. Markers are for B cells (CD20), T cells (CD3D), fibroblasts (aSMA), macrophages (CD68), and other living cells (Hoechst DNA stain).

**Methods:**

This relationship elicited an in-depth characterization of these granuloma-associated immune structures using recently developed spatial biology techniques, such as high-plex tissue immunofluorescence imaging and spatial transcriptomics. The approach used combined classical histology (hematoxylin and eosin staining) with spatial profiling of protein antigens and mRNA by multiplexed cyclic immunofluorescence (CyCIF) and GeoMx Digital Spatial Profiler, respectively, from 3 TB-infected tissue samples. A companion computational framework, MCMICRO, transformed multi-channel whole-slide images into single-cell data for subsequent analysis.

**Figure:**
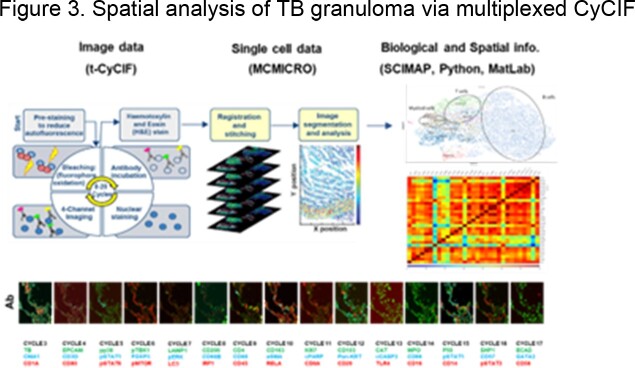

Iterative rounds of multi-channel immunofluorescence are performed, followed by computational analysis on MCMICRO to yield a stitched multiplexed image and single-cell data, which can be analyzed in SCIMAP

**Figure:**
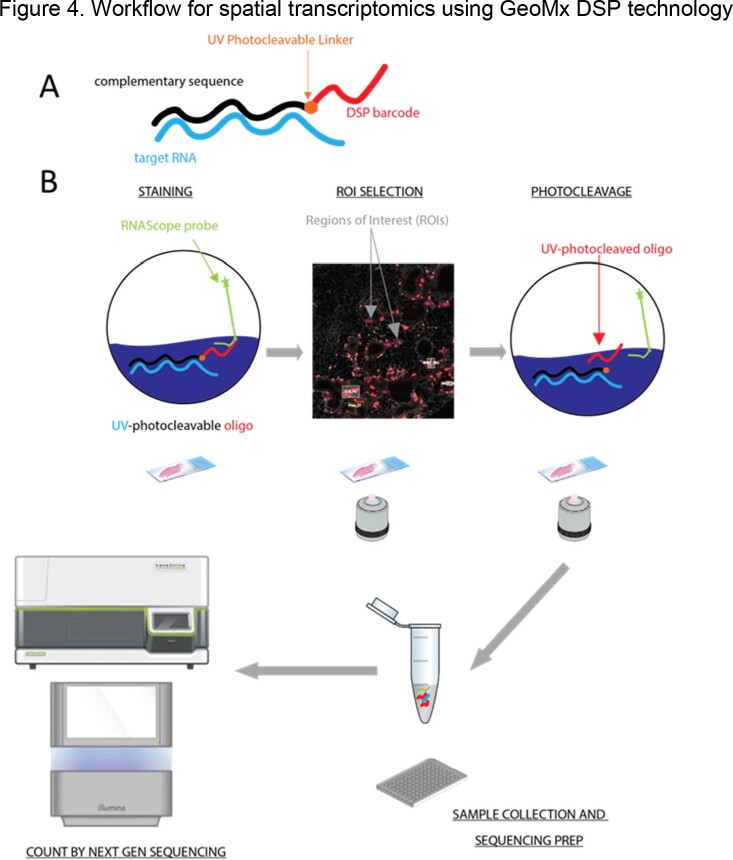
A. An oligonucleotide DNA barcode is attached to an in-situ probe for mRNA detection, with a photocleavable linker. B. GeoMx workflow

**Results:**

Unsupervised analysis of the CyCIF protein marker expression levels in all the TLSs by nonnegative matrix factorization (NMF) suggests 2 or 3 "types" of TLSs with distinct marker co-occurrence patterns. Similarly, analysis of the whole transcriptome by GeoMx reveals two classes of TLSs.

**Figure:**
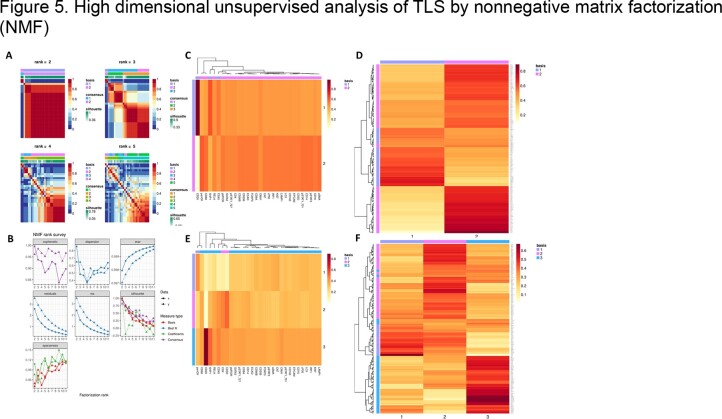

Median expression of 30 CyCIF protein markers (expressed by cells within each TLS) were subjected to the NMF algorithm. A. Predicted classification and effectiveness of each classification scheme plotted as a heatmap. B. NMF parameters with 2- and 3- type classification showing highest probabilities for real data (solid line). Marker importance and clustering for 2-type (C, D) and 3-type (E, F) classification.

**Figure:**
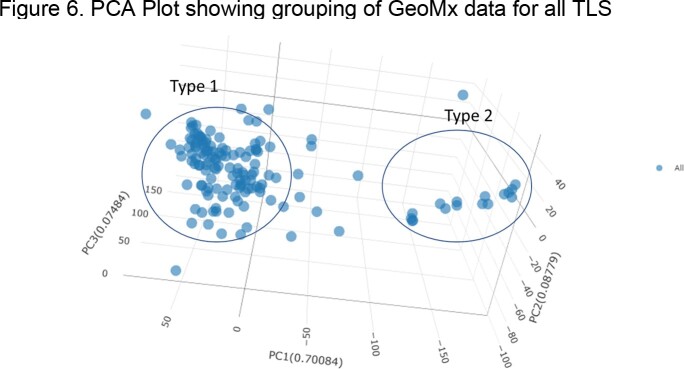

**Conclusion:**

As the granuloma lesions were annotated by histology prior to spatial analysis, it was not evident that there was a proximate relationship between granuloma pathology and the TLS type they are associated with. Future work is needed to expound on differential immune pathways associated with these TLS types and how they relate to TB granuloma biology.

**Disclosures:**

**All Authors**: No reported disclosures

